# Post-progression survival after cessation of treatment with nivolumab for advanced non-small cell lung cancer: A retrospective study

**DOI:** 10.1371/journal.pone.0203070

**Published:** 2018-08-28

**Authors:** Yukihiro Yano, Hiroyuki Kurebe, Ryuya Edahiro, Yuki Hosono, Saeko Nakatsubo, Kohei Nishida, Nobuyuki Sawa, Mikako Ishijima, Takeshi Uenami, Masaki Kanazu, Yuki Akazawa, Toshihiko Yamaguchi, Masahide Mori

**Affiliations:** Department of Thoracic Oncology, National Hospital Organization Toneyama National Hospital, Toyonaka, Osaka, Japan; Baylor College of Medicine, UNITED STATES

## Abstract

**Objectives:**

The effectiveness of treatment after cessation of nivolumab in patients with advanced non-small cell lung cancer (NSCLC) has not been well investigated. The aim of the present study was to clarify the clinical benefit of post-nivolumab treatment in such patients.

**Materials and methods:**

A retrospective review was conducted on patients who received treatment after cessation of nivolumab due to disease progression or adverse events at the Toneyama National Hospital between January 2016 and April 2017.

**Results:**

Among 64 patients treated with nivolumab, 26 patients received treatment after cessation of nivolumab due to disease progression (n = 21) or adverse events (n = 5). The median age of the patients was 68 years and 19 patients were male. Nineteen patients had performance status (PS) 1 or less at initiation of post-nivolumab treatment. Four, 20, and 2 patients were treated with platinum doublets, a single agent, and molecular targeting agents, respectively. Response rate, disease control rate, and median progression-free survival of first-line post-nivolumab treatment were 34.6% (9 patients), 73.1% (19 patients), and 2.8 months (95% confidence interval [CI]: 1.7–5.2), respectively. Adverse events (≥ grade 3) and treatment cessation were observed in 57.7% (15 patients) and 19.2% (5 patients), respectively. There were no statistically significant differences for the majority of patient characteristics between the groups with (n = 26) and without post-nivolumab treatment. However, PS at cessation of nivolumab and post-progression survival (PPS) after cessation of nivolumab (median PPS: 12.6 vs. 1.4 months, 95% CI: 3.8–14.7 vs. 0.4–2.2) were significantly different between the groups. A multivariate Cox regression analysis showed significant correlation of PS at cessation of nivolumab (hazard ratio [HR]: 0.34, 95% CI: 0.13–0.87) and post-nivolumab treatment (HR: 0.19, 95% CI: 0.08–0.43) with prolonged PPS after nivolumab.

**Conclusion:**

Median post-progression survival in patients with advanced NSCLC who received post-nivolumab treatment was approximately 1 year.

## Introduction

Lung cancer is one of the leading causes of mortality worldwide. Cytotoxic chemotherapy has been the standard treatment of this disease for decades. Molecular targeting agents such as gefitinib, one of the epidermal growth factor receptor (EGFR) tyrosine kinase inhibitors (TKIs), became available one and half decade ago. The introduction of EGFR-TKIs in clinical practice changed the strategy for the treatment of non-small cell lung cancer (NSCLC). Nowadays, other molecular targeting agents such as anaplastic lymphoma kinase (ALK)-TKIs have also become available. In recent years, the novel mechanism of immune checkpoint inhibitors (ICIs), that differs from conventional immunotherapies, has received great attention. Programmed cell death 1 (PD-1) inhibitors block a signal preventing activated T cells from attacking cancer cells. Nivolumab is the first PD-1 inhibitor approved in many countries for the treatment of NSCLC. Numerous pivotal studies showed a survival benefit of treatment with nivolumab in patients with NSCLC [1, 2]. Pembrolizumab, another PD-1 inhibitor, has also shown a similar survival benefit to nivolumab [3]. Furthermore, the efficacy of pembrolizumab as first-line therapy in NSCLC patients with high programmed death ligand 1 (PD-L1) expression has been reported [4]. These results emphasized the importance of PD-1 inhibitors in the treatment of lung cancer and drastically altered the therapeutic strategy against this disease.

Nevertheless, more than half of NSCLC patients treated with a PD-1 inhibitor fail their treatment and require subsequent therapy. Recently, Schvartsman *et al*. reported a higher overall response rate to single-agent chemotherapy after immunotherapy compared with that observed in historical data from the pre-PD-1 inhibitor era [5]. Moreover, a Korean study recently reported increased response rates to salvage chemotherapy administered after treatment with a PD-1 inhibitor [6]. However, there are currently no data available regarding the treatment of NSCLC after failure of a PD-1 inhibitor regimen. The effectiveness of treatment in patients with advanced NSCLC after cessation of treatment with nivolumab due to disease progression or adverse events has not been well investigated. Therefore, the present study was conducted to assess the clinical benefit of post-nivolumab treatment in such patients.

## Materials and methods

### Patients

A retrospective review was conducted using medical records of patients who received treatment after cessation of nivolumab due to disease progression or adverse events at the Toneyama National Hospital between January 2016 and April 2017. Nivolumab was administered based on the clinical decision by the attending physician. Dosage and schedule of nivolumab administration were 3mg per body weight and once every 2 weeks. Data collected by the end of August 2017 were used for analysis. Post-progression survival (PPS) was defined as the survival time following progressive disease (PD) during treatment with nivolumab. In the present study, definition of partial response, stable disease, and PD were based on the RECIST ver. 1.1. However, for patients in whom the RECIST criteria could not be applied, those were determined clinically. Objective response rate was defined as the ratio of patients with partial response to all study patients. Disease control rate was defined as the ratio of patients with partial response and stable disease to all study patients. Progression-free survival (PFS) and PPS were calculated from the date of initiation of post-nivolumab treatment, and the date of diagnosis of PD during treatment with nivolumab, respectively. The present study was approved by the Toneyama National Hospital Institutional Review Board (Approval number: 1701). This approval allowed for retrospective chart review and anonymous reporting of the results without requiring patient informed consent.

### Statistical methods

All comparisons were performed using the JMP version 8 statistical software package (SAS Institute; Cary, NC, USA). Variables are expressed as means ± standard deviation or as median and range. Categorical values are expressed as numbers and percentages. Percentages are expressed in relation to the total population unless otherwise specified. Comparisons were performed using the Student’s t test for continuous variables, and Fisher’s exact test for categorical variables. Survivals were assessed using the Kaplan Meier method and determined by the log-rank test. In addition, a multivariate Cox regression model was used to examine factors that may influence survival after cessation of nivolumab administration. For all analyses, a value of p<0.05 indicated a statistically significant difference.

## Results

### Patients

**Fig 1:**
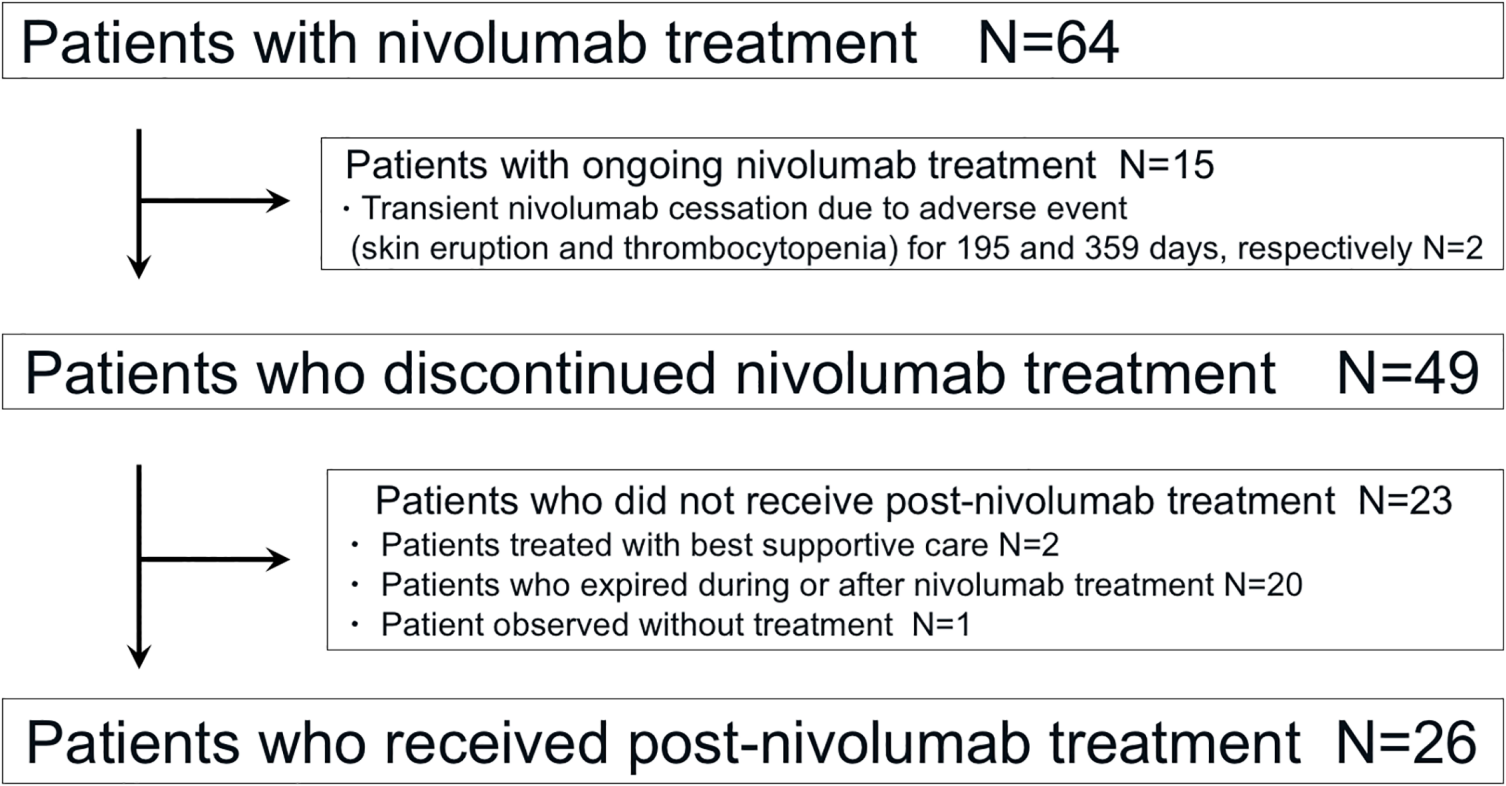
Flow chart of patient selection.

Fig 1 shows the flow chart of patient selection. Among 64 patients who received treatment with nivolumab, 49 patients discontinued treatment. Of those who discontinued, 23 patients did not receive further treatment, and the remaining 26 patients received treatment after cessation of nivolumab administration due to disease progression (n = 21) or adverse events (n = 5). Patient characteristics are shown in Table 1. The median age of patients was 68 years (range: 42–84 years), and 19 patients were male. The most common Eastern Cooperative Oncology Group (ECOG) performance status (PS) at initiation of post-nivolumab treatment was 1, observed in 14 patients. Histologically, 8 patients had squamous cell carcinoma of the lung and 17 patients had non-squamous cell carcinoma. Five patients had driver mutations in their lung cancer. The median number of regimens prior to treatment with nivolumab was 2 (range: 1–10). Thoracic and brain radiotherapy prior to treatment with nivolumab were performed in 7 and 9 patients, respectively. The median cycles of treatment with nivolumab were 4.5 (range: 1–15). Progressive disease and non-PD, as the best response to treatment with nivolumab, were observed in 15 and 11 patients, respectively.

**Table 1:** Patient characteristics.

Variables	N = 26
Gender, N (%)	
Male	19 (73.1%)
Female	7 (26.9%)
Age, years	
Median (range)	68 (42–84)
ECOG performance status at initiation of post-nivolumab treatment, N (%)	
0	5 (19.2%)
1	14 (53.8%)
2	6 (23.1%)
3	1 (3.8%)
Smoking status, N (%)	
Ex-smoker	21 (80.8%)
Never	5 (19.2%)
Brinkman Index, Mean ± SD	568.1 ± 452.4
Histology, N (%)	
Non-Sq.	17 (65.4%)
Sq.	8 (30.8%)
Non-small	1 (3.8%)
Driver mutations, N (%)	
Yes	5 (19.2%)
No	20 (76.9%)
Unknown	1 (3.8%)
Number of regimens prior to nivolumab, N (%)	
Median (range)	2 (1–10)
≦1	12 (46.2%)
≧ 2	14 (53.8%)
Thoracic RT prior to nivolumab, N (%)	
Yes	7 (26.9%)
No	19 (73.1%)
Brain RT prior to nivolumab, N (%)	
Yes	9 (34.6%)
No	17 (65.4%)
Cycles of nivolumab	
Median (range)	4.5 (1–15)
Best response to nivolumab, N (%)	
Non-PD	11 (42.3%)
PD	15 (57.7%)
Cessation of nivolumab due to adverse events, N (%)	
Yes	5 (19.2%)
No	21 (80.8%)

ECOG = Eastern Cooperative Oncology Group, SD = standard deviation, Sq = squmanous cell carcinoma of the lung, Non-Sq = non squmanous cell carcinoma of the lung, RT = radiotherapy, PD = progressive disease

### Treatment

**Fig 2:**
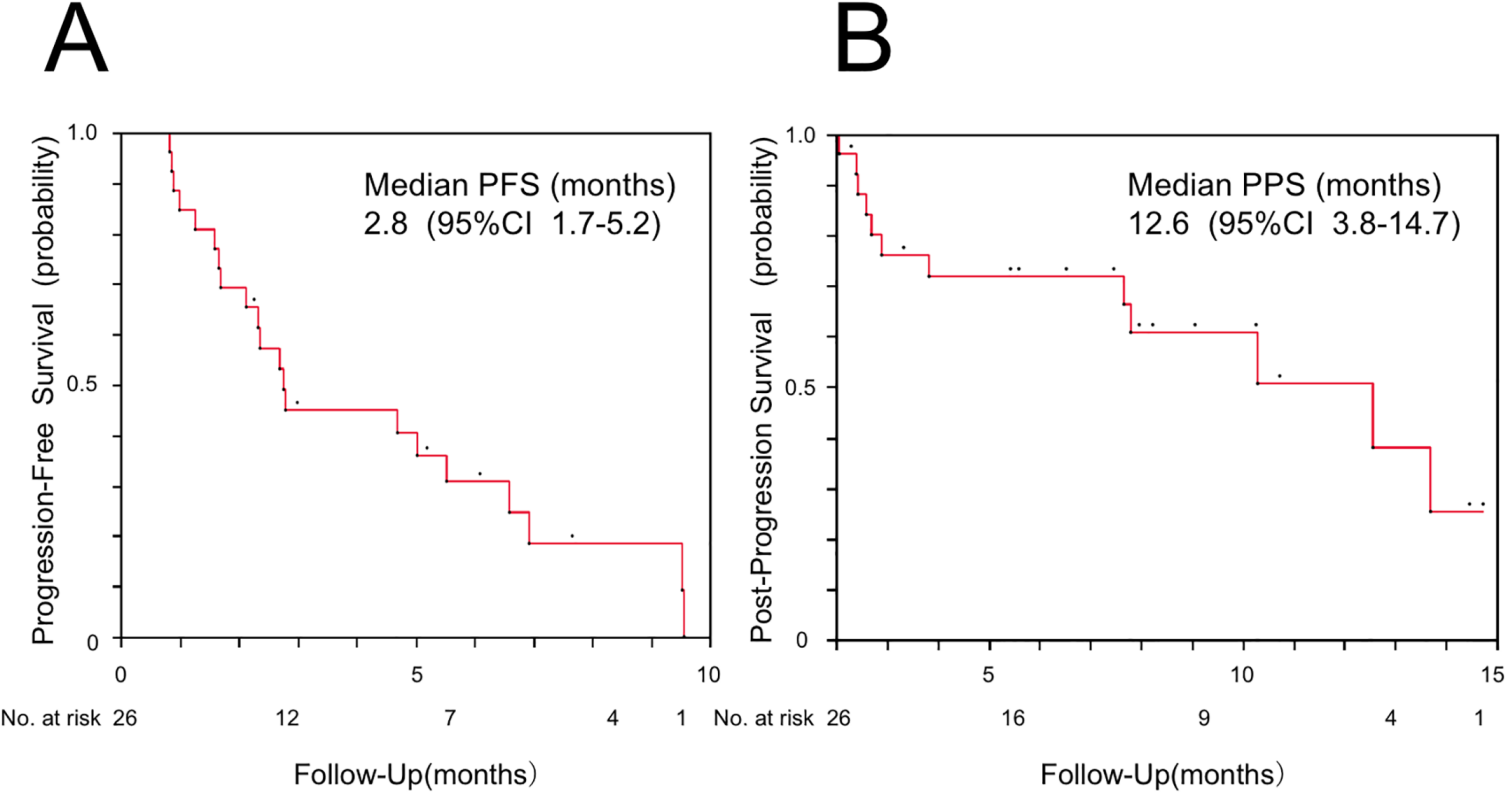
Kaplan-Meier plot. (A) progression-free survival (PFS) of first-line post-nivolumab treatment and (B) post-progression survival (PPS) after cessation of treatment with nivolumab in 26 patients who received post-nivolumab chemotherapy. The PFS was calculated from the date of initiation of first-line post-nivolumab treatment and the PPS was calculated from the date of diagnosis of progressive disease during nivolumab treatment. CI; confidence interval.

**Fig 3:**
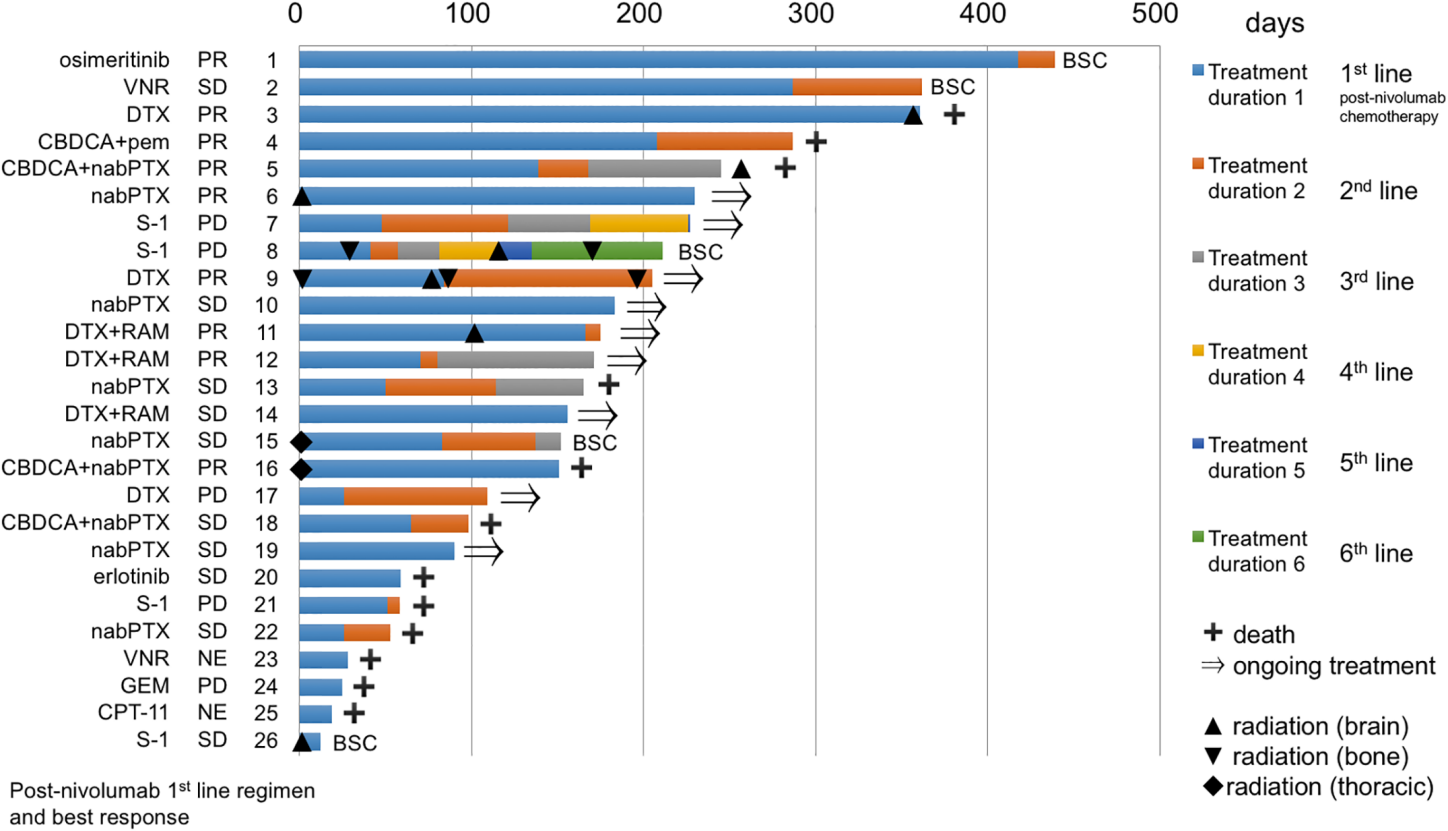
Course of treatment for each patient. Each bar indicates treatment duration for each patient. Treatment regimens, best response to each treatment, and patient numbers are described on the left side of each bar. On the right side of each bar, the status of each patient is shown. Diamond and triangle shapes represent radiation therapy in each patient. VNR; vinorelbine, DTX; docetaxel, CBDCA; carboplatin, pem; pemetrexed, nabPTX; nab-paclitaxel, RAM; ramucirumab, GEM; gemcitabine, CPT-11; irinotecan, SD; stable disease, PR; partial response, PD; progressive disease, NE; not evaluable; BSC; best supportive care.

The results of first-line post-nivolumab treatment are shown in Table 2. The overall response rate and disease control rate were 34.6% (9 patients) and 73.1% (19 patients), respectively. Radiotherapy was performed in 8 patients after cessation of treatment with nivolumab. Adverse events (≥ grade 3) were observed in 57.7% (15 patients), and treatment cessation occurred in 19.2% (5 patients). The efficacy and safety profile of each treatment regimen are shown in Table 3. Platinum doublets regimens and regimens which included taxane without platinum were administered in 4 and 12 patients, respectively. Molecular targeting agents such as erlotinib and osimertinib were also administered (1 patient each). The response rates to taxane regimens without platinum were 41.7% (5 patients). The median PFS of first-line post-nivolumab treatment was 2.8 months (95% confidence interval [CI]: 1.7–5.2 months) (Fig 2A). The median PPS after cessation of treatment with nivolumab was 12.6 months (95% CI: 3.8–14.7 months) (Fig 2B). The course of treatment for each patient is shown in Fig 3. Radiotherapy for intrathoracic lesions prior to first-line post-nivolumab chemotherapy was performed in 2 patients (Patient 15 and 16). The maximum number of post-nivolumab treatment regimens was 6 (Patient 8).

**Table 2:** Treatment results.

Variables	N = 26
Cycles of treatment	
Median (range)	2 (1–11)
Effectiveness of treatment	
Complete response, N (%)	0 (0%)
Partial response, N (%)	9 (34.6%)
Stable disease, N (%)	10 (38.5%)
Progressive disease, N (%)	5 (19.2%)
Not evaluable, N (%)	2 (7.7%)
Objective response rate	34.6%
Disease control rate	73.1%
Radiotherapy	
Thoracic, N (%)	2 (7.7%)
Brain, N (%)	5 (26.9%)
Brain + bone, N (%)	2 (7.7%)
Adverse events	
Neutropenia grade 4, N (%)	4 (15.4%)
Neutropenia grade 3, N (%)	7 (26.9%)
Anemia grade 4, N (%)	1 (3.8%)
Anemia grade 3, N (%)	2 (7.7%)
Thrombocytopenia grade 3, N (%)	1 (3.8%)
Liver dysfunction grade 3, N (%)	1 (3.8%)
Diarrhea grade 3, N (%)	1 (3.8%)
Pneumonitis grade 2, N (%)	1 (3.8%)
Anaphylaxis, N (%)	1 (3.8%)
≧grade 3 adverse events, N (%)	15 (57.7%)
Cessation of treatment due to adverse event, N (%)	5 (19.2%)

**Table 3:** Result of each treatment regimens.

Regimens	N = 26	Cycles median (range)	Response	Disease control	Radiation	≧grade 3 Adverse events
Carboplatin + nab-paclitaxel	3 (11.5%)	3 (2–6)	2 / 3 (66.7%)	3 / 3 (100%)	thoracic 1, brain 1	3 / 3 (100%)
Carboplatin + pemetrexed	1 (3.8%)	9	1 / 1 (100%)	1 / 1 (100%)		1 / 1 (100%)
Platinum doublets	4 (15.4%)		3 / 4 (75%)	4 / 4 (100%)		
nab-paclitaxel	6 (23.1%)	2.5 (1–8)	1 / 6 (16.7%)	6 / 6 (100%)	thoracic 1, brain 1	1 / 6 (16.7%)
Docetaxel + ramucirumab	3 (11.5%)	4 (2–8)	2 / 3 (66.7%)	3 / 3 (100%)	brain 1	2 / 3 (66.7%)
Docetaxel	3 (11.5%)	4 (2–11)	2 / 3 (66.7%)	2 / 3 (66.7%)	brain 1, brain + bone 1	2 / 3 (66.7%)
Taxanes	12 (46.2%)		5 / 12 (41.7%)	11 / 12 (91.7%)		
S-1	4 (15.4%)	1 (1–1)	0 / 4 (0%)	1 / 4 (25%)	brain 1, brain + bone 1	2 / 4 (50%)
Vinorelbine	2 (7.7%)	6 (2–10)	1 / 2 (50%)	1 / 2 (50%)		2 / 2 (100%)
Irinotecan	1 (3.8%)	1	not evaluable	not evaluable		1 / 1 (100%)
Gemcitabine	1 (3.8%)	1	0 / 1 (0%)	0 / 1 (0%)		1 / 1 (100%)
Erlotinib	1 (3.8%)	81 days	1 / 1 (100%)	1 / 1 (100%)		1 / 1 (100%)
Osimertinib	1 (3.8%)	286 days	1 / 1 (100%)	1 / 1 (100%)		0 / 1 (0%)
Tyrosine kinase inhibitors	2 (7.7%)		2 / 2 (100%)	2 / 2 (100%)		

### Comparison between patients with and without post-nivolumab treatment

**Fig 4:**
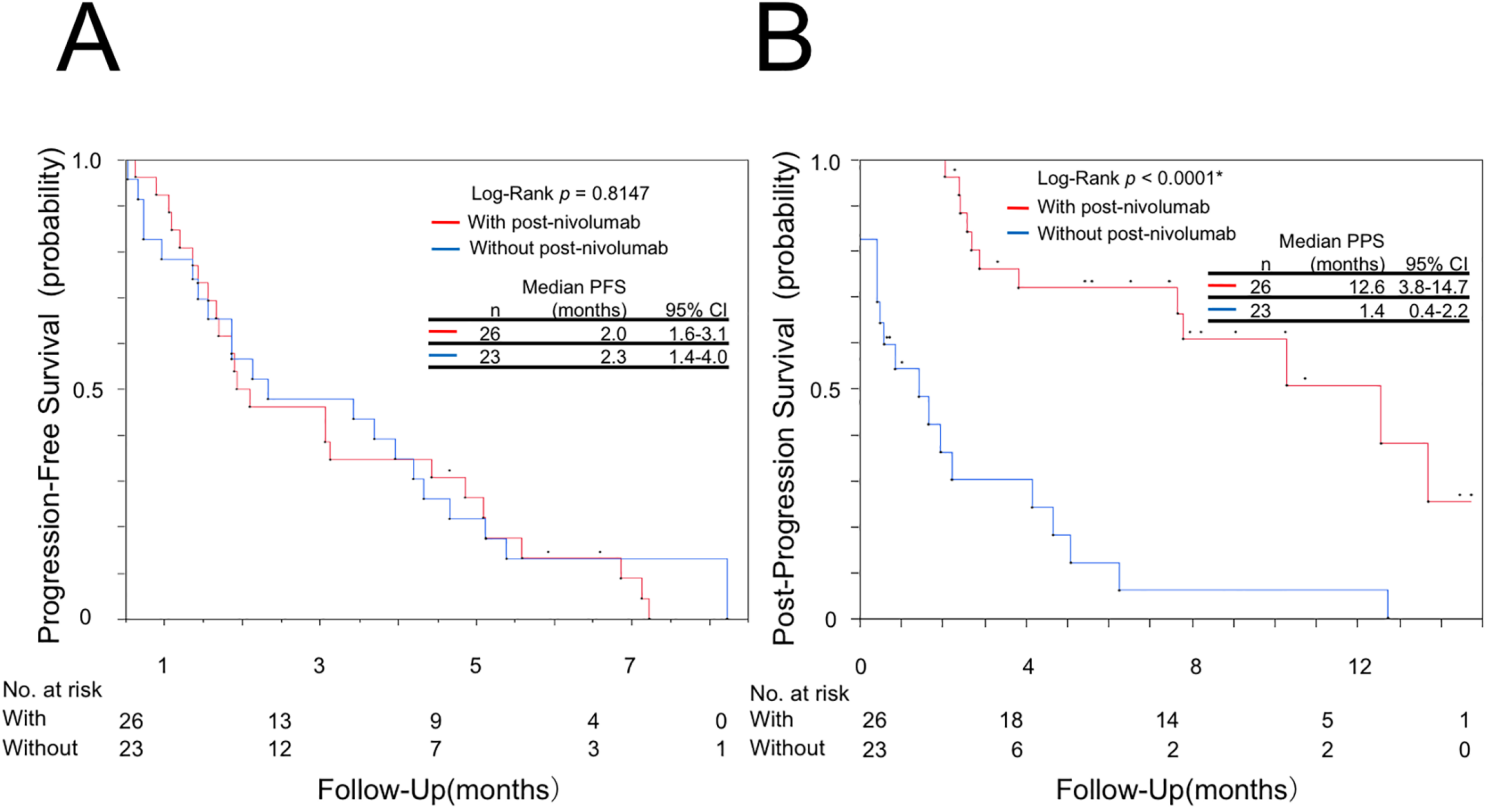
Kaplan-Meier plot. (A) progression-free survival (PFS) of first-line post-nivolumab treatment and (B) post-progression survival (PPS) after cessation of treatment with nivolumab in 26 patients who received post-nivolumab chemotherapy (red line), and 23 patients who did not receive post-nivolumab chemotherapy (blue line). CI; confidence interval.

Characteristics of patients with and without post-nivolumab treatment are shown in Table 4. There were no statistically significant differences observed regarding the majority of patient characteristics between these two groups. Similarly, no statistically significant differences were observed regarding PFS between the two groups (Fig 4A). However, PS at cessation of treatment with nivolumab (p = 0.0102) and median PPS after cessation of treatment with nivolumab (p<0.0001) were significantly different between the two groups (Fig 4B). Subsequently, a multivariate analysis demonstrated that PS at cessation of treatment with nivolumab (hazard ratio [HR]: 0.34, 95% CI: 0.13–0.87, p = 0.0249) and post-nivolumab treatment (HR: 0.19, 95% CI: 0.08–0.43, p<0.0001) correlated significantly with prolonged PPS after cessation of treatment with nivolumab (Table 5).

**Table 4:** Differences between patients with or without post-nivolumab treatment.

Post-nivolumab treatment	With N = 26	Without N = 23	p value
Gender, N (%)			
Male	19 (73.1%)	16 (69.6%)	1
Female	7 (26.9%)	7 (30.4%)	
Age, years			
Mean ± SD	67.0 ± 10.1	69.3 ± 10.1	0.4372
Smoking status*, N (%)			
Ex-smoker	21 (80.8%)	17 (77.3%)	1
Never	5 (19.2%)	5 (22.7%)	
Brinkman Index			
Mean ± SD	568.1 ± 452.4	701.2 ± 557.8	0.3711
Histology**, N (%)			
Non-Sq.	17 (68.0%)	16 (69.6%)	1
Sq.	8 (32.0%)	7 (30.4%)	
Driver mutations***, N (%)			
Yes	5 (20.0%)	4 (17.4%)	1
No	20 (80.0%)	19 (82.6%)	
ECOG performance status at initiation of post-nivolumab treatment, N (%)			
≦1	23 (88.5%)	17 (73.9%)	0.2732
≧ 2	3 (11.5%)	6 (26.1%)	
Number of regimens prior to nivolumab, N (%)			
Mean ± SD	3.3 ± 3.0	2.4 ± 2.0	0.2209
≦1	12 (46.2%)	10 (43.5%)	1
≧ 2	14 (53.8%)	13 (56.5%)	
Thoracic RT prior to nivolumab, N (%)			
Yes	7 (26.9%)	3 (13.0%)	0.2689
No	19 (73.1%)	20 (87.0%)	
Brain RT prior to nivolumab, N (%)			
Yes	9 (34.6%)	12 (52.2%)	0.3944
No	17 (65.4%)	11 (47.8%)	
Cycles of nivolumab			
Mean ± SD	5.6 ± 3.7	5.0 ± 3.6	0.6135
Best response to nivolumab****, N (%)			
Non-PD	11 (42.3%)	9 (40.9%)	1
PD	15 (57.7%)	13 (59.1%)	
Cessation of nivolumab due to adverse events, N (%)			
Yes	5 (19.2%)	4 (17.4%)	1
No	21 (80.8%)	19 (82.6%)	
ECOG performance status at cessation of treatment with nivolumab, N (%)			
≦1	19 (73.1%)	8 (34.8%)	0.0102*
≧ 2	7 (26.9%)	15 (65.2%)	

SD = standard deviation, Sq = squmanous cell carcinoma of the lung, Non-Sq = non squmanous cell carcinoma of the lung, ECOG = Eastern Cooperative Oncology Group, RT = radiotherapy, PD = progressive disease. The smoking status*, histology**, driver mutations*** and best response to nivolumab**** could not be determined in 1 patient without post-nivolumab treatment, 1 patient with post-nivolumab treatment, 1 patient with post-nivolumab treatment, and 1 patient without post-nivolumab treatment, respectively.

**Table 5:** Univariate and multivariate analyses of clinical factors influencing PPS after cessation of nivolumab.

Variables	HR	Univariate 95% CI	*p* value	HR	Multivariate 95% CI	*p* value
Gender						
Male vs Female	1.96	0.86–5.05	0.111			
Age						
≦69 vs. ≧ 70	0.78	0.38–1.67	0.518			
Smoking status						
Ex-smoker vs. Never	1.95	0.77–6.05	0.166			
Histology						
Non-Sq. vs. Sq.	0.82	0.39–1.85	0.622			
Driver mutations						
Yes vs. No	0.52	0.17–1.31	0.173			
ECOG performance status at initiation of nivolumab						
≦1 vs. ≧ 2	0.4	0.18–0.96	0.042*	0.63	0.23–1.77	0.371
Number regimens prior to nivolumab						
≦1 vs. ≧ 2	1.19	0.57–2.61	0.650			
Thoracic RT prior to nivolumab						
Yes vs. No	0.61	0.18–1.58	0.331			
Brain RT prior to nivolumab						
Yes vs. No	1.47	0.72–3.00	0.286			
Cycles of nivolumab						
≦4 vs. ≧ 5	1.84	0.87–4.24	0.112			
Best response to nivolumab						
Non-PD vs. PD	0.72	0.32–1.52	0.4			
ECOG performance status at cessation of nivolumab						
≦1 vs. ≧ 2	0.21	0.09–0.46	<0.0001*	0.34	0.13–0.87	0.0249*
Post-nivolumab treatment						
Yes vs. No	0.17	0.08–0.37	<0.0001*	0.19	0.08–0.43	<0.0001*

PPS = post-progression survival, HR = hazard ratio, CI = confidence interval, Sq = squmanous cell carcinoma of the lung, Non-Sq = non squmanous cell carcinoma of the lung, ECOG = Eastern Cooperative Oncology Group, RT = radiotherapy, PD = progressive disease

## Discussion

The treatment strategy after cessation of PD-1 inhibitor administration against NSCLC has emerged as an important clinical issue. Currently, there are limited data available to overcome this challenge.

The present study showed that treatment response to first-line post-nivolumab chemotherapy in patients with advanced NSCLC, treated after cessation of treatment with nivolumab, were higher compared with that obtained from historical data with conventional chemotherapy as late-line treatment. Furthermore, median PPS after cessation of treatment with nivolumab in patients with advanced NSCLC who received post-nivolumab chemotherapy was approximately 1 year. Moreover, this median PPS was significantly longer in patients treated with post-nivolumab chemotherapy than in untreated patients regardless of the results of the best response and PFS of treatment with nivolumab (Table 4 and Fig 4A). We speculate these results suggest whether primary or acquired resistance to nivolumab after initial response to nivolumab may not affect the results of post-nivolumab treatment.

Recently, Schvartsman et al. reported that the response rate to single-agent chemotherapy after immunotherapy was higher than that obtained from historical data from the pre-PD-1 inhibitor era. They surveyed 26 patients retrospectively and demonstrated a response rate of 39% [5]. This was the first study to investigate the effectiveness of chemotherapy after failure of treatment with PD-1 inhibitor and suggested that PD-1 inhibitors may confer delayed synergism to subsequent cytotoxic therapy. Moreover, studies from the United States have suggested additional effects of cytotoxic therapy after treatment with PD-1 inhibitor [7, 8]. Recently, a Korean study demonstrated increased response rates to salvage chemotherapy administered after treatment with PD-1 inhibitor. The overall response rate to salvage chemotherapy administered after immunotherapy was significantly higher than that to last chemotherapy administered before immunotherapy (53.4% vs. 34.9%; p = 0.03). The investigators concluded that treatment with PD-1 inhibitors may render tumors more vulnerable to subsequent chemotherapy [6]. Other studies showed that conventional chemotherapy as second-line or later treatment is associated with relatively low response rates (10%-20%) against advanced NSCLC. Yoshioka et al. compared docetaxel with amrubicin, a third-generation anthracycline and a potent topoisomerase II inhibitor, in 202 patients with previously treated NSCLC. The study was conducted before the era of PD- inhibitor usage in Japan and thus, the response rates are not influenced by treatment with a PD-1 inhibitor. The overall response rate was 19.6% versus 14.4% in the docetaxel and amrubicin groups, respectively [9]. In the present study, the overall response rate was similar (34.6%) to the rates reported in the aforementioned American and Korean studies, and higher than the rate reported by Yoshioka et al.

A recent Japanese study investigated PPS after treatment failure with EGFR-TKIs such as gefitinib or erlotinib. Survival time of patients treated with second-line chemotherapy after cessation of treatment with EGFR-TKIs was 13.9 months [10]. These patients were considered to have relatively good prognosis compared with patients without driver mutations. In the present study, PPS was 12.6 months and 76.9% of patients did not have driver mutations, with all patients receiving post-nivolumab chemotherapy as third-line or later treatment. Furthermore, more than half of patients received fourth-line therapy or later (Table 1). In the pre-PD-1 inhibitor era, prognosis after failure of first- or second-line treatment was considered poor. The present study is the first to indicate a median PPS of approximately one year in patients with advanced NSCLC, treated with chemotherapy after cessation of treatment with nivolumab. These PPS data may suggest a survival benefit of cytotoxic chemotherapy in these patients.

Immune checkpoint inhibitors combined with chemotherapy have shown high efficacy as first-line treatment against advanced NSCLC. The objective response rates for nivolumab 10 mg/kg plus gemcitabine-cisplatin, nivolumab 10 mg/kg plus pemetrexed-cisplatin, nivolumab 10 mg/kg plus paclitaxel-carboplatin, and nivolumab 5 mg/kg plus paclitaxel-carboplatin were 33%, 47%, 47%, and 43%, respectively [11]. The objective response rates for pembrolizumab plus pemetrexed-carboplatin was 55% [12]. Combination therapy with a PD-1 inhibitor and cytotoxic chemotherapy is a promising treatment strategy for NSCLC. Langer et al. described the rationale of this combination therapy as antitumor activity of cytotoxic chemotherapy mediated through a direct cytotoxic effect, as well as an immunological effect i.e. the reduction of T-regulatory cell activity and enhancement of tumor antigen cross-presentation [12]. Chemotherapy after treatment with a PD-1 inhibitor may act as combination therapy due to the prolonged binding of PD-1/PD-L1 receptor by the PD-1 inhibitor. One infusion of nivolumab (3 mg/kg) indicated approximately 70% of PD-1 receptor occupancy for at least 60 days. Repeated infusion of nivolumab (10 mg/kg) maintained ≥50% PD-1 receptor occupancy for approximately 200 days [13]. PD-1 inhibitors binding the PD-1/PD-L1 receptor may exert additional effect to that of cytotoxic chemotherapeutic agents. Preclinical data suggest that cytotoxic chemotherapeutic agents have direct cytotoxic effects to cancer cells as well as immunomodulatory effects [14]. Platinum agents downregulate PD-L2 expression in human dendritic cells resulting in enhanced antigen-specific proliferation and Th1 cytokine secretion, as well as enhanced recognition of tumor cells by T cells [15]. Therefore, platinum-based anticancer agents may enhance the immune-stimulatory potential of dendritic cells and decrease the immunosuppressive capability of tumor cells. Docetaxel may deplete regulatory T cells and alter the expression of interferon-*γ* and transforming growth factor-*β* to improve antitumor immunity [16]. The administration of cisplatin plus vinorelbine to NSCLC patients appears to significantly increase the ratio between effector and regulatory T cells and reduce immunosuppressive activity in the majority of patients [17]. These preclinical data, together with the results of the present study, suggest that the synergistic effect of PD-1 inhibitors and cytotoxic chemotherapy may confer a higher response to chemotherapy and prolonged survival after treatment failure with PD-1 inhibitors.

The limitations of the present study must be acknowledged. Firstly, although the treatment response was assessed based on the RECIST, the interval of radiographic examination was not uniform among patients. Thus, the response rate and PFS could not be determined accurately. Secondly, 2 patients received radiation therapy for intrathoracic lesions prior to initiation of post-nivolumab chemotherapy and this radiation therapy may affect the response to subsequent chemotherapy. Thirdly, the smoking status, histology, and driver mutations could not be determined in 1 patient without post-nivolumab treatment, 1 patient with post-nivolumab treatment, and 1 patient with post-nivolumab treatment, respectively. Therefore, these patients were excluded from statistical analysis. Finally, this was a retrospective study with a small sample size. Further studies with larger sample size are warranted to verify the findings presented herein and ensure successful application to clinical practice.

In conclusion, the median post-progression survival in patients with advanced NSCLC, treated with chemotherapy after cessation of nivolumab administration was approximately 1 year. These data on PPS suggest a possible survival benefit of cytotoxic chemotherapy in these patients.

## References

[1] Brahmer J, Reckamp KL, Baas P, Crinò L, Eberhardt WE, Poddubskaya E, et al Nivolumab versus docetaxel in advanced squamous-cell non-small-cell lung cancer. N Engl J Med. 2015; 373: 123–135. 10.1056/NEJMoa1504627 26028407 PMC4681400

[2] Borghaei H, Paz-Ares L, Horn L, Spigel DR, Steins M, Ready NE, et al Nivolumab versus docetaxel in advanced nonsquamous non-small-cell lung cancer. N Engl J Med. 2015; 373: 1627–1639. 10.1056/NEJMoa1507643 26412456 PMC5705936

[3] Herbst RS, Baas P, Kim DW, Felip E, Pérez-Gracia JL, Han JY, et al Pembrolizumab versus docetaxel for previously treated, PD-L1-positive, advanced non-small-cell lung cancer (KEYNOTE-010): a randomised controlled trial. Lancet. 2016; 387: 1540–1550. 10.1016/S0140-6736(15)01281-7 26712084

[4] Reck M, Rodríguez-Abreu D, Robinson AG, Hui R, Csőszi T, Fülöp A, et al Pembrolizumab versus chemotherapy for PD-L1-positive non-small-cell lung cancer. N Engl J Med. 2016; 375: 1823–1833. 10.1056/NEJMoa1606774 27718847

[5] Schvartsman G, Peng SA, Bis G, Lee JJ, Benveniste MFK, Zhang J, et al Response rates to single-agent chemotherapy after exposure to immune checkpoint inhibitors in advanced non-small cell lung cancer. Lung Cancer. 2017; 112: 90–95. 10.1016/j.lungcan.2017.07.034 29191606

[6] Park SE, Lee SH, Ahn JS, Ahn MJ, Park K, Sun JM. Increased response rates to salvage chemotherapy administered after PD-1/PD-L1 inhibitors in patients with non-small cell lung cancer. J Thorac Oncol. 2018; 13: 106–111. 10.1016/j.jtho.2017.10.011 29101058

[7] Leger PD, Rothschild SI, Castellanos EH, Pillai RN, York SJ, Horn L, et al Salvage chemotherapy following exposure to immune checkpoints inhibitors in patients with NSCLC. J Clin Oncol. 2017; 35: Abstract 9084.

[8] Grigg C, Reuland BD, Sacher AG, Yeh R, Rizvi NA, Shu CA. Clinical outcomes of patients with non-small cell lung cancer (NSCLC) receiving chemotherapy after immune checkpoint blockade. J Clin Oncol. 2017; 35: Abstract 9082.

[9] Yoshioka H, Katakami N, Okamoto H, Iwamoto Y, Seto T, Takahashi T, et al A randomized, open-label, phase III trial comparing amrubicin versus docetaxel in patients with previously treated non-small-cell lung cancer. Ann Oncol. 2017; 28: 285–291. 10.1093/annonc/mdw621 28426104

[10] Kogure Y, Saka H, Oki M, Saito TI, Ahmed SN, Kitagawa C, et al Post-progression survival after EGFR-TKI for advanced non-small-cell lung cancer harboring EGFR mutations. PLoS One. 2015 8 11;10(8):e0135393 10.1371/journal.pone.0135393 eCollection 2015. 26262682 PMC4532435

[11] Rizvi NA, Hellmann MD, Brahmer JR, Juergens RA, Borghaei H, Gettinger S, et al Nivolumab in combination with platinum-based doublet chemotherapy for first-line treatment of advanced non-small-cell lung cancer. J Clin Oncol. 2016; 34: 2969–2979. 10.1200/JCO.2016.66.9861 27354481 PMC5569693

[12] Langer CJ, Gadgeel SM, Borghaei H, Papadimitrakopoulou VA, Patnaik A, Powell SF, et al Carboplatin and pemetrexed with or without pembrolizumab for advanced, non-squamous non-small cell lung cancer: a randomised, phase 2 cohort of the open-label KEYNOTE-021 study. Lancet Oncol. 2016; 17: 1497–1508. 10.1016/S1470-2045(16)30498-3 27745820 PMC6886237

[13] Brahmer JR, Drake CG, Wollner I, Powderly JD, Picus J, Sharfman WH, et al Phase I study of single-agent anti-programmed death-1 (MDX-1106) in refractory solid tumors: safety, clinical activity, pharmacodynamics, and immunologic correlates. J Clin Oncol 2010; 28: 3167–3175. 10.1200/JCO.2009.26.7609 20516446 PMC4834717

[14] Galluzzi L, Buqué A, Kepp O, Zitvogel L, Kroemer G. Immunological effects of conventional chemotherapy and targeted anticancer agents. Cancer Cell. 2015; 28: 690–714. 10.1016/j.ccell.2015.10.012 26678337

[15] Lesterhuis WJ, Punt CJ, Hato SV, Eleveld-Trancikova D, Jansen BJ, Nierkens S, et al Platinum-based drugs disrupt STAT6-mediated suppression of immune responses against cancer in humans and mice. J Clin Invest. 2011; 121: 100–108. 10.1172/JCI43656 PMC3148725 21765211

[16] Li JY, Duan XF, Wang LP, Xu YJ, Huang L, Zhang TF, et al Selective depletion of regulatory T cell subsets by docetaxel treatment in patients with nonsmall cell lung cancer. J Immunol Res. 2014;2014:286170. 10.1155/2014/286170 Epub 2014 Apr 28. 24868562 PMC4020463

[17] Roselli M, Cereda V, di Bari MG, Formica V, Spila A, Jochems C, et al Effects of conventional therapeutic interventions on the number and function of regulatory T cells. Oncoimmunology. 2013 10 1;2(10):e27025 10.4161/onci.27025 24353914 PMC3862634

